# Understanding how traumatic brain injury-related changes in fluid biomarkers affect quality of life outcomes in veterans: a prospective observational trial protocol (UNTANGLE)

**DOI:** 10.1136/bmjopen-2024-084818

**Published:** 2024-08-19

**Authors:** Youstina Metry, Christel McMullan, Rachel Upthegrove, Antonio Belli, Renata S M Gomes, Richard J Blanch, Zubair Ahmed

**Affiliations:** 1 Institute of Inflammation and Ageing, University of Birmingham, Birmingham, UK; 2 Ophthalmology Department, Queen Elizabeth Hospital Birmingham, Birmingham, UK; 3 Institute of Applied Health Research, University of Birmingham, Birmingham, UK; 4 Centre for Patient Reported Outcomes Research, University of Birmingham Institute of Applied Health Research, Birmingham, West Midlands, UK; 5 Centre for Human Brain Health, University of Birmingham, Birmingham, West Midlands, UK; 6 Institue for Mental Health, University of Birmingham, Birmingham, UK; 7 University Hospitals Birmingham NHS Foundation Trust, Birmingham, UK; 8 Department of Nursing, Midwifery and Health, Northumbria University, Newcastle upon Tyne, UK; 9 Academic Department of Military Surgery and Trauma, Royal Centre for Defence Medicine, Birmingham, UK

**Keywords:** Brain Injuries, Quality of Life, MOLECULAR BIOLOGY, Neuro-ophthalmology, Patient Reported Outcome Measures, Depression & mood disorders

## Abstract

**Introduction:**

Traumatic brain injury (TBI) is a major cause of disability, with annual global incidence estimated as 69 million people. Survivors can experience long-term visual changes, altered mental state, neurological deficits and long-term effects that may be associated with mental illness. TBI is prevalent in military personnel due to gunshot wounds, and blast injury. This study aims to evaluate the relationship between evolving visual, biochemical and mental health changes in both military veterans and civilians, suffering from TBI, and detect preliminary indicators of prognosis for TBI recovery, and quality-of-life outcomes.

**Methods and analysis:**

UNTANGLE is a 24-month prospective observational pilot study recruiting three patient groups: civilians with acute moderate-severe TBI, military veterans with diagnosis of a previous TBI and a control group of civilians or veterans with no history of a previous TBI. Patients will undergo visual, biochemical and mental health assessments, as well as patient-reported quality of life outcome measures over the course of a 1-year follow-up period.

**Ethics and dissemination:**

Ethical approval has been obtained from the Health Research Authority and Health and Care Research Wales with a REC reference number of 23/NW/0203. The results of the study will be presented at scientific meetings and published in peer-reviewed journals, including both civilian and military-related publications. We will also present our findings at national and international meetings of learnt neuroscience and neuropsychiatry and military societies. We anticipate that our pilot study will inform a larger study on the long-term outcomes of TBI and quality of life, specific to military veterans, such that potential interventions may be accessed as quickly as possible.

**Trial registration number:**

ISRCTN13276511https://doi.org/10.1186/ISRCTN13276511.

**STRENGTHS AND LIMITATIONS OF THIS STUDY**

- This is a prospective study measuring visual and serum biomarkers, mental health and quality of life symptoms within two different cohorts of traumatic brain injury (TBI) patients (acute TBI in civilians and in veterans with a diagnosis of previous TBI) for comparison.
- Inclusion of an acute civilian TBI group will ensure that we will be able to capture patients within 14 days after a TBI, meaning that early predictors and pathogenic events will be captured.
- Veterans may only be included in the study some months after the TBI, due to access to this population, possibly introducing confounding variables.
- As a pilot study, the number of participants in each group will be small, limiting the generalisability of the results. Nonetheless, our pilot study will generate critical data for the design and sample size estimations of future trials.

## Introduction

Head injury is the most common cause of death and disability in people <40 years old in the UK, with 1.4 million people a year attending emergency departments in England and Wales, and about 200 000 people requiring hospital admission.^1^ Five per cent of injuries are moderate to severe traumatic brain injury (TBI), which can have fatal outcomes and long-term disability. The most common cause of the injury in the elderly is falls in contrast to road traffic collisions in the young.^2^ TBI, whether mild (mTBI) or severe (sTBI) results in neurological deficits as well as altered cognitive or mental state, contributing to the increase in mental health disorders that are seen post-TBI.^3 4^ For example, depression,^4^,^8^ obsessive compulsive disorders,^9^,^13^ post-traumatic stress disorder (PTSD) and post-traumatic amnesia,^14^,^16^ psychosis,^8^ and personality changes such as apathy^7^ and aggression^8^ can occur after TBI, and increases the burden of living with TBI.^14^

According to the TRACK-TBI study, one in five individuals experience a mental health disorder after mTBI,^14 16^ and the rates of completed suicide after TBI were almost three times the general population in these patients.^16^ TBI is a particular concern in military personnel, exposed to events such as gunshot wounds or explosive blast injury. Of military personnel returning from Iraq and/or Afghanistan, up to 40% of personnel in the USA had a mTBI,^17^ and 25% in the UK cohort,^18^ with one report suggesting that mental health and neurological disorders could affect as many as 70% of veterans.^19^ Individuals sustaining TBI can also develop optic nerve, tract, radiation or occipital lobe damage that manifest as colour vision, stereopsis and visual field defects, with corresponding retinal changes detected by optical coherence tomography (OCT).^20^,^24^

Up to 80% of TBI patients suffer visual disturbances. Given that structural changes to the optic nerve manifest post-TBI, including in subclinical patients, it is possible that changes in visual structure and function may have future uses in diagnosis and prognostication of TBI, as well as being important to the patient’s function and well-being.^20 25^ It is recognised that individuals with visual impairment are at greater risk of developing mental health problems such as depression and anxiety.^26^ For example, nearly one-third of individuals with visual impairment experienced mild depressive symptoms, while 10%–45% of people reported moderate to severe depressive symptoms,^27^,^29^ and since vision loss is treated as a physical condition, the psychological impact is often under-recognised.^30^

Since there is an overlap between brain injury and mental health disorders, which may occur after damage to neuronal pathways, TBI fluid and imaging biomarkers may have diagnostic and prognostic value for TBI severity and mental health outcomes. For example, the fluid biomarkers glial fibrillary acidic protein (GFAP), S100β, tumour necrosis factor-α and myelin basic protein are associated with TBI severity, and have been suggested as biomarkers of mental health disorders with poor prognosis.^31^

Currently, TBI treatments are limited, and long-term interventions are symptomatic. Therefore, a better understanding of the neuropsychiatric changes caused by TBI, through longitudinal monitoring of biofluids and visual function along with patient-reported quality of life measures in acute TBI patients and military veterans with TBI, could provide insights into potential preventative treatments after TBI and possible disease-specific and transdiagnostic discovery bioscience with significant and wider impact. The study could therefore inform the design of a much-needed larger clinical trial in this patient population.

### Study hypotheses

1. TBI-induced alterations in brain structure are associated with the risk of subsequent mental health disorder.
2. TBI-induced changes can be detected using fluid and imaging biomarkers.
3. TBI-induced changes in biomarkers may identify those at risk of poor recovery and the development of mental health conditions affecting the quality of life.
4. Changes in visual structure and function predict mental health and quality of life and associate with TBI-related biomarkers.

#### Study aims

To conduct a pilot study on the long-term outcomes of TBI and correlate this with candidate biomarkers in blood and saliva from veterans and non-veterans with TBI alongside measures of quality-of-life measures such as visual and mental health assessments and patient-reported outcome measures (PROMs). Through these multidimensional assessments, the study aims to better understand the relationship between the different long-term manifestations of TBI and how quality of life is affected by the injury.

### Methods and analysis

#### Overall study design

**Figure 1:**
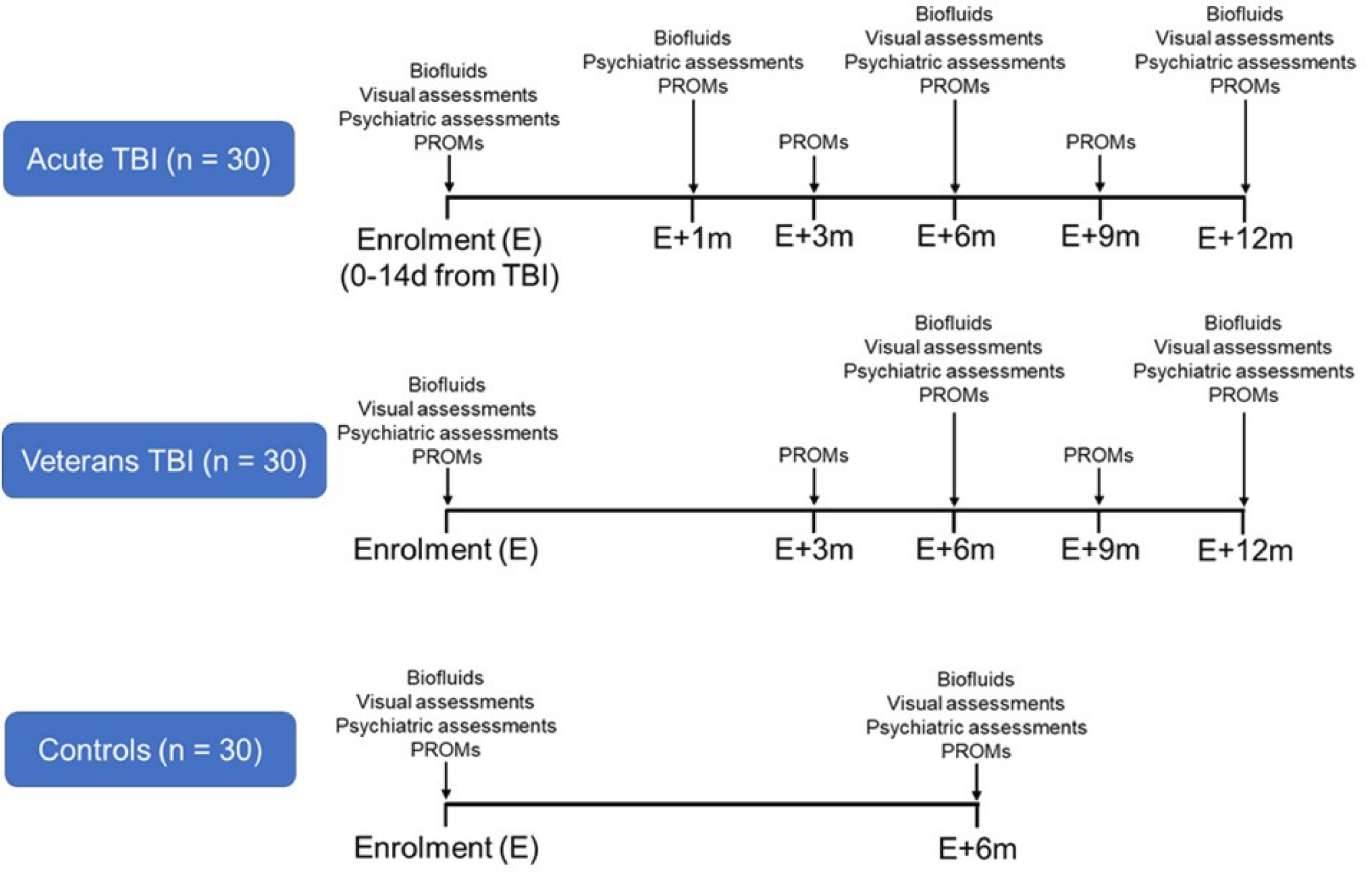
Biofluids, visual, psychiatric assessments and PROMs timeline. PROMs, patient-reported outcome measures; TBI, traumatic brain injury.

This is a single centre prospective observational pilot study in three groups of 30 participants each: civilians admitted to the Queen Elizabeth Hospital (QEH) in Birmingham with acute moderate-severe TBI, military veterans with diagnosis of a previous TBI and a control group of civilians or veterans with no history of TBI. Patients will undergo visual, biochemical and mental health assessments, as well as patient-reported symptoms and quality of life outcome measures over the course of a 1-year follow-up period (see figure 1 for overview).

In the acute TBI group (predictive group), biofluid collection, visual function, depression, anxiety, PTSD, suicidal behaviour, alcohol use and quality of life data through patient-reported outcome measures (PROMs) will be collected within 14 days after TBI. The same tests will be repeated in outpatients at 6- and 12 months after enrolment. At 1 month after enrolment, biofluid collection, psychiatric symptoms and quality of life outcomes will be repeated to monitor the recovery trajectory. PROMs will be completed remotely by each participant every 3 months for the 12-month follow-up period.

Veterans will also have biofluids collected, visual function and psychiatric assessments and quality of life measures collected using PROMs at enrolment and at 6- and 12-month follow-up. PROMs will also be completed remotely by participants every 3 months.

Control participants will be age and gender-matched to the acute TBI group and recruited from both civilian and veterans cohorts with no TBI history. They will have biofluids collected, visual function and psychiatric assessments and quality of life measures collected via PROMs at enrolment and at 6 months only (see tables1^3^ for planned assessment schedules in the three participant groups).

**Table 1:** Assessments delivered within the trial period for acute traumatic brain injury patient group

	Trial period			
	Enrolment	Initial assessment (may be at enrolment)	Postallocation (monitoring interval clinically determined)	Close-out
Timepoint	−t_1_	Contact 1 (0–14 days post head injury or at enrolment)	Contact 2–5 (1, 3, 6 and 9 months after head injury or enrolment)	Contact 6 (12 months after head injury)
Enrolment				
Eligibility screen	X			
Informed consent	X			
Assessments				
Collect blood and saliva		X(face to face)	X(1 and 6 months only, face to face)	X(face to face)
Visual function tests (visual acuity, visual field, optical coherence tomography (OCT), OCT angiography, pupillometry, saccadic latency, autorefraction, colour vision and contrast sensitivity)		X(face to face)	X(1 and 6 months only, face to face)	X(face to face)
Social and occupational functioning assessment		X(face to face)	X(1 and 6 months only, face to face)	X(face to face)
Self-reported outcomes measures collected by post or electronically (eg, BIVSS visual impairment, PHQ-9, GAD-7, PCL-5, SBQ-R, AUDIT and EQ-5D-5L)		X(all collected remotely)	X(1, 3, 6 and 9 months, all collected remotely)	X(all collected remotely)
Head injury outcome				X

AUDITAlcohol Use Disorders Identification TestBIVSSBrain Injury Vision Symptom SurveyEQ-5D-5LEuroQol 5-Dimension 5-Level (Quality of Life)GAD-7General Anxiety Disorder-7PCL-5Post-Traumatic Stress Disorder Checklist for DSM-5PHQ-9Patient Health Questionnaire-9SBQ-RSuicidal Behaviour Questionnaire Revised

**Table 2:** Assessments delivered within the trial period for the veteran’s participant group

	Trial period			
	Enrolment	Initial assessment (may be at enrolment)	Postallocation (monitoring interval clinically determined)	Close-out
Timepoint	−t_1_	Contact 1 (at enrolment)	Contact 2–4 (3, 6 and 9 months after enrolment)	Contact 5 (12 months after head injury)
Enrolment				
Eligibility screen	X			
Informed consent	X			
Assessments				
Collect blood and saliva		X(face to face)	X(at 6 months only, face to face)	X(face to face)
Visual function tests (visual acuity, visual field, optical coherence tomography (OCT), OCT angiography, pupillometry, saccadic latency, autorefraction, colour vision and contrast sensitivity)		X(face to face)	X(at 6 months only, face to face)	X(face to face)
Social and occupational functioning assessment		X(face to face)	X(at 6 months only, face to face)	X(face to face)
Self-reported outcomes measures collected by post or electronically (BIVSS visual impairment, PHQ-9, GAD-7, PLC-5, SBQ-R, AUDIT and EQ-5D-5L)		X(all collected remotely)	X(at 3, 6 and 9 months, all collected remotely)	X(all collected remotely)
Head injury outcome				X

AUDITAlcohol Use Disorders Identification TestBIVSSBrain Injury Vision Symptom SurveyEQ-5D-5LEuroQol 5-Dimension 5-Level (Quality of Life)GAD-7General Anxiety Disorder-7PCL-5Post-Traumatic Stress Disorder Checklist for DSM-5PHQ-9Patient Health Questionnaire-9SBQ-RSuicidal Behaviour Questionnaire Revised

**Table 3:** Assessments delivered within the trial period for healthy control group

	Trial period	
	Initial assessment (may be at enrolment)	Postallocation (monitoring interval clinically determined)
Timepoint	Contact 1 (single assessment point)	Contact 2 (6 months after enrolment)
Enrolment		
Eligibility screen	X	
Informed consent	X	
Assessments		
Collect blood and saliva	X(face to face)	X(face to face)
Visual function tests (visual acuity, visual field, optical coherence tomography (OCT), OCT angiography, pupillometry, saccadic latency, autorefraction, colour vision and contrast sensitivity)	X(face to face)	X(face to face)
Social and occupational functioning assessment	X(face to face)	X(face to face)
Self-reported outcomes measures collected by post (BIVSS visual impairment, PHQ-9, GAD-7, PLC-5, SBQ-R, AUDIT and EQ-5D-5L)	X(all collected remotely)	X(6 months, all collected remotely)
Head injury outcome	N/A	

AUDITAlcohol Use Disorders Identification TestBIVSSBrain Injury Vision Symptom SurveyEQ-5D-5LEuroQol 5-Dimension 5-Level (Quality of Life)GAD-7General Anxiety Disorder-7PCL-5Post-Traumatic Stress Disorder Checklist for DSM-5PHQ-9Patient Health Questionnaire-9SBQ-RSuicidal Behaviour Questionnaire Revised

The acute TBI cohort will enable us to investigate changes related to recent TBI. This will be investigated only within the civilian patients due to the restricted access to military personnel sustaining recent TBI. Veterans cohort will be included within the study, enabling investigating the longer-term effects on TBI. We, accordingly, will be able to evaluate findings in participants with TBI history against the control group over a long-period of time.

The study opened recruitment in December 2023 and based on the current recruitment pace, we estimate that the last participant will continue until December 2025.

#### Eligibility and exclusion criteria

To be included in the study, participants are required to be over 18 years old, have two eyes and must have sustained TBI. Control participants should not have a diagnosis or history of moderate or severe TBI. Exclusion criteria for all three groups include: participants below 18 years old, are pregnant, have pre-existing neuropsychiatric conditions, retinal or optic nerve disorder or are registered as sight impaired.

#### Outcome measures

Outcome measures will include fluid biomarkers in blood and saliva, visual functions tests, social and occupational functioning assessments, and self-reported quality of life outcomes such as Brain Injury Vision Symptom Survey visual impairment, Patient Health Questionnaire-9, General Anxiety Disorder-7, Post-Traumatic Stress Disorder Checklist for DSM-5, Suicidal Behaviour Questionnaire Revised, Alcohol Use Disorders Identification Test and EuroQol 5-Dimension 5-Level (Quality of Life) (tables4^7^).

**Table 4:** Potential fluid biomarkers

Biomarker	Description	Reference
Glial fibrillar acidic protein (GFAP)	Astroglial injury marker	37 40
S100β	Astroglial injury marker	^37 38 41^
Neuron-specific enolase (NSE)	Neuronal injury marker	^42 43^
Neurofilament light chain (NF-L)	Neuronal injury marker	^37 39 40^
Ubiquitin C-terminal hydrolase-L1 (UCLH-1)	Neuronal injury marker	^37 39 40^
Phosphorylated tau (pTau)	Neuronal injury marker	^37 39 40^
Myelin basic protein (MBP)	Neuronal injury marker, myelin disruption	^44 45^
Tumour necrosis factor-α (TNF-α), interleukin-6 (IL-6) and IL1-β	Inflammation	^45^
Cortisol	Stress response protein	^46^

**Table 5:** miRNA biomarkers

Biomarker	Description	Reference
miR-20a-5p	Neuronal injury marker	^47^
miR-24-3 p	Neuronal injury marker	47 49
miR-27b-3p	Recovery process monitoring	^48^
miR-29c-3p	Correlate with burden of head impacts and neuronal development overtime since injury	^50 51^
miR-181a-5p	Associated with protein modifications in traumatic brain injury	^48 52^

**Table 6:** Visual function assessments

Test used	Visual function assessed	References
Acuity Plus package on the Colour Assessment and Diagnosis machine (CAD; City Occupational, London, UK)	Visual acuity, colour vision and contrast	^20^
Humphrey visual field testing using the 24-2 SITA-Fast technique (Carl Zeiss, Cambridge, UK)	Visual field	^20 53^
Email package on the CAD	Saccadic latency	^20 54^
Autorefraction (Rexxam, Tokyo, Japan)	Accommodation	^20 54^
Pupillometry (DP-2000, Neuro-optics California, USA)	Pupillary reactions	2055 57
Optic coherence tomography (OCT; Heidelberg Spectralis, Heidelberg Engineering, Heidelberg, Germany) using Posterior Pole, retinal nerve fibre layer, and disc volume images	Retinal thickness	^58^
OCT angiography (OCTA), HS-20 scanning protocol.	Retinal blood flow	^58^

**Table 7:** Patient-reported outcomes assessed and scales

Outcome assessed	Scale used	Reference
Brain injury-induced vision symptoms	BIVSS (Brain Injury Vision Symptom Survey)	^59^
Depression	PHQ-9 (Patient Health Questionnaire)	^60^
Anxiety	GAD-7 (General Anxiety Disorder)	^61^
Post-traumatic stress disorder (PTSD)	PCL-5 (Checklist for DSM-5)	^62^
Suicide ideation	SBQ-R (Suicidal Behaviour Questionnaire Revised)	^63^
Alcohol abuse	AUDIT (Alcohol Use Disorders Identification Test)	^64 65^
Quality of life	EQ-5D-5L (Quality of Life)	^64 65^

#### Fluid biomarkers

Fluid biomarkers from blood will look for a panel of biomarkers, summarised in table 4, that detect ongoing astroglial and neuronal injury/damage such as GFAP and neuron-specific enolase. Salivary biomarkers will look for dysregulated micro RNAs after TBI, including miR20a-5p and miR-24-3p (table 5).

#### Visual function tests

Visual function tests are summarised in table 6.

#### Mental health and quality of life outcome measures

Quality of life outcomes such as visual impairment, depression, anxiety, PTSD, suicide ideation, alcohol use disorder and alcohol abuse and overall quality of life symptoms will be assessed using validated questionnaires, summarised in table 7.

In addition, clinician-led social occupation and functioning (SOFAS) test will be conducted at each face-to-face visit,^32^ while head injury outcomes using the Glasgow Coma Scale^33 34^ and the Mayo-Portland Adaptability Inventory^35^ will be assessed at the final visit as part of routine care by a physician.

#### Statistical methods

Analyses will include all available data of recruited patients (not restricted to patients with complete follow-up). Between group comparisons will evaluate susceptibility to poor mental health and quality of life outcomes. Longitudinal data may show the latency of onset of mental health and quality of life problems after acute TBI, in order to time future interventions.

Candidate serum and visual biomarkers will be compared between groups and associated with mental health and quality of life outcomes to look for predictive value. Many of these data are continuous and normally distributed and repeated measures data (including data from both right and left eyes). As such, generalised estimating equations will be used to determine changes over time with data from each eye and different time points treated as repeated measures, with a binomial distribution used for binary and ordinal outcomes as appropriate. Trial statisticians in the Birmingham Clinical Trials Team will be engaged for further statistical advice, if required.

This is a pilot study to evaluate longitudinal changes in fluid and visual biomarkers of brain injury, mental health status and self-reported quality of life measures in patients with TBI (civilian and military veterans). As such, sample size is based on:

1. Statistical advice that in a pilot/feasibility study, 30 subjects in each group are recommended to allow estimation of the parameters of interest.
2. Childs *et al*^36^ who showed a difference of 5 µm in global retinal nerve fibre layer (RNFL) thickness between boxers and controls, an average RNFL thickness of 101 and an SE of 1.8.

Past experience in the concussion clinic suggests that we can follow 80% of patients at the 3-month point (ie, only 20% lost to follow-up). We powered the study to detect a 5 µm change in the RNFL thickness, assuming a correlation of 0.9 between repeated measures (conservative given that in the absence of pathology, RNFL thickness varies very little). Simulated power calculations in R revealed that 24 participants with complete data (recruiting 30 and assuming a 20% drop out) would have >80% power to detect a 2 µm change in RNFL thickness and 100% power to detect a 5 µm decrease.

### Study limitations

It is important to point out that there are several limitations of this study. For example, some patients may be unable to commit to all of the study follow-up visits and hence there is a risk that patients might withdraw from the study resulting in potentially low enrolment numbers. Adherence to the follow-up visits is also difficult to ensure and is an inherent problem in all clinical trials and especially in pilot trials of this nature that recruit low numbers. Among the veteran population, there may be different times from their TBI to enrolment in this study and this may confound some of the outcome measures.

### Patient and public involvement

Patients and/or public were not involved in the design of this protocol.

### Ethics and dissemination

#### Research ethics approval

The study involves human participants and ethical approval was approved by the UK Health Research Agency through Northwest Haydock Research Ethics Committee (REC reference of 23/NW/0203).

### Consent

Within both veteran and control groups, participants should have capacity to consent and be willing and able to follow the protocol, except for the acute TBI cohort, who may not have capacity to consent as a result of TBI. Written consent will be obtained by the local research team, which may include the site principal investigator, co-investigators or research assistants. Veterans will be recruited through veteran charities such as Bravo Victor, Blind Veterans UK or other veterans charities and organisations. In the acute TBI groups attending the QEH in Birmingham for head injury, identified participants may not have the capacity to consent and as such, written consultation will be obtained from a personal (ie, relative) or nominated (professional) consultee. However, if a patient regains consciousness and capacity, their consultee will be notified, and the patient will be given the opportunity to provide written consent to remain in the study or withdraw. Unconscious patients will only have blood collected and OCT scans conducted as all other tests require a patient to be conscious and have capacity to complete. The willingness of participants to remain in the study will be re-affirmed at each study visit with the patient or consultee as appropriate. Patients have the right to withdraw from the study at any time, but all data collected will be retained and used in the analysis to avoid bias in assessment of outcomes.

### Data access

The final deidentified dataset will be made fully accessible on reasonable request once the results are published.

### Dissemination policy

The results from this study will be disseminated through our University’s website (http://www.bham.ac.uk) and through open access publication in high impact journals, including both civilian and military publications. We will also present our findings at national and international meetings of learned neuroscience, neuropsychiatry, and military societies.
