# Supplementary material for: Understanding how traumatic brain injury-related changes in fluid biomarkers affect quality of life outcomes in veterans: a prospective observational trial protocol (UNTANGLE)
Source: BMJ Open. 2024 Aug 19;14(8):e084818. doi: 10.1136/bmjopen-2024-084818 (PMC11337664; doi:10.1136/bmjopen-2024-084818)
Supplement: Supplementary file 1 [file bmjopen-2024-084818.reviewer_comments.pdf]

PEER REVIEW HISTORY

BMJ Open publishes all reviews undertaken for accepted manuscripts. Reviewers are asked to complete a checklist review form and are provided with free text boxes to elaborate on their assessment. These free text comments are reproduced below.

ARTICLE DETAILS

**TITLE (PROVISIONAL)**	Understanding how Traumatic Brain Injury-related changes in fluid biomarkers affect quality of life outcomes in veterans: a prospective observational trial protocol (UNTANGLE)
**AUTHORS**	Metry, Youstina; McMullan, Christel; Upthegrove, Rachel; belli, antonio; Gomes, Renata S.M.; Blanch, Richard; Ahmed, Zubair

VERSION 1 - REVIEW

**REVIEWER NAME**	Lindblad, Caroline
**REVIEWER AFFILIATION**	Karolinska Institute, Clinical Neuroscience
**REVIEWER CONFLICT OF INTEREST**	The reviewer is a participant in a long-term follow-up study concerning (exclusively civilian) severe TBI (first author Friberg et al 2022, published as a conference abstract: https://www.liebertpub.com/doi/10.1089/neu.2022.29128.abstracts). The follow-up time-line and overall study design in this work is different than that of the study under review.
**DATE REVIEW RETURNED**	11-Mar-2024

**GENERAL COMMENTS**	Overall:<br>This is an ambitious pilot study that is currently recruiting. The authors aim to recruit 90 patients in total from a control cohort, a civilian, and a veteran TBI cohort, respectively. Although there are some methodological questions that could be clarified, the overall work is clear and succinct with clear research hypotheses and aims. Moreover, there is a robust ethical section, where consideration has been taken to the vulnerability of the study cohort. Taken together, this seems as an important pilot study for unraveling long-term predictors following moderate-severe TBI among veterans. My recommendation therefore is accept.<br><br>Manuscript<br>Title: the abbreviation UNTANGLE is not intuitively linked to the rest of the title<br><br>Abstract:<br>- Please clarify: 69 million world-wide is what you intend?<br><br>Introduction:<br>- Interesting, but the link between mental health and visual disturbance could be clarified<br><br>Aim<br>- This is a pilot study on long-term outcomes of TBI and quality of life, specific to military veterans<br><br>Methods<br>- Project status: recruiting. Dates: Dec 2023 – fall 2025.<br>- Aim: to recruit n = 90 patients in total (30 patients per group)

	- Time-of-inclusion is different across the study cohorts. The authors acknowledge this as an important limitation.<br>- It is unclear how matching of patients will be done across the study groups. Age-matched? Propensity score-matched?<br><br>BMJ provides reviewers with a guide for review items, which the authors have fulfilled.

**REVIEWER NAME**	Tsoukalas, Dimitris
**REVIEWER AFFILIATION**	Metabolomic Medicine
**REVIEWER CONFLICT OF INTEREST**	None
**DATE REVIEW RETURNED**	29-Apr-2024

**GENERAL COMMENTS**	The manuscript "UNTANGLE: Understanding how TBI-related changes in fluid<br>biomarkers affect quality of life outcomes in veterans: a prospective observational trial protocol" presents the study design to detrmine potential biomarkers associated with vision impairement, QoL parameters and metal health in individuals subbering from traumatic brain injury.<br><br>A strength of the study is that it is a prospective cohort study and the reported outcomes cover a wide variety of test.<br>However it is not clear how the authors have selected the specific groups, what confounding factors they might need to consider.<br>For example are the samples going to be age-matched, because the veterans group might be older than the two other groups. Also is there a limitation for veternas depending on the time between TBI incident and enrollement?<br>In addition, are they going to be gender-matched?<br>Also it is better to clarify whether the control group is from the general population or veteran with no TBI as this affects the research question.<br><br>Also, the authors should consider analyzing potenital biomarkers depending on TBI stage and their progression with time.<br><br>In addition, how are you going to ensure adherence, adress low enrollment and other methodological problems?<br><br>In addition, it would be interesting to look for neurotransmitters changes and discuss their potential clinical value<br>https://www.nature.com/articles/s41380-018-0239-6<br><br><br>Overall, the study covers an interesting topic though the study protocol lacks important details

VERSION 1 – AUTHOR RESPONSE

Reviewer: 1

Comment 1: This is an ambitious pilot study that is currently recruiting. The authors aim to recruit 90 patients in total from a control cohort, a civilian, and a veteran TBI cohort, respectively. Although there are some methodological questions that could be clarified, the overall work is clear and succinct with clear research hypotheses and aims. Moreover, there is a robust ethical section, where consideration

has been taken to the vulnerability of the study cohort. Taken together, this seems as an important pilot study for unraveling long-term predictors following moderate-severe TBI among veterans. My recommendation therefore is accept.
Author response: Thank you for your time in reviewing our manuscript.

Comment 2: Title: the abbreviation UNTANGLE is not intuitively linked to the rest of the title
Author response: We believe UNTANGLE is a good acronym for the study and reflects our wish to untangle how TBI changes in biomarkers may predict quality of life. Furthermore, the study has been approved by the funders using this acronym and registered with the Health Research Authority and ISRCT with this acronym and hence we are reluctant to try to change this although we have changed the order of the acronym in the study title to "Understanding how Traumatic Brain Injury-related changes in fluid biomarkers affect quality of life outcomes in veterans: a prospective observational trial protocol (UNTANGLE)"

Comment 3: Abstract: - Please clarify: 69 million world-wide is what you intend?
Author response: Indeed, our apologies. This has been clarified.

Comment 4: Introduction: - Interesting, but the link between mental health and visual disturbance could be clarified.
Author response: The link between vision loss and mental health problems has been clarified further, see Lines 100-111 in the marked copy and 100-106 in the clean copy.

Comment 5: Aim - This is a pilot study on long-term outcomes of TBI and quality of life, specific to military veterans
Author response: We have clarified this further in Lines136-142 in the marked copy and 132-138 in the clean copy. The study also follows non veterans and hence the outcomes are not just specific to veterans but is applicable to civilians.

Comment 6: Methods- It is unclear how matching of patients will be done across the study groups. Age-matched? Propensity score-matched?
Author response: This has now been clarified in Lines 162-163 in the marked copy and 158-159 in the clean copy .

Reviewer: 2
Comment 1: A strength of the study is that it is a prospective cohort study and the reported outcomes cover a wide variety of test. However it is not clear how the authors have selected the specific groups, what confounding factors they might need to consider.
For example are the samples going to be age-matched, because the veterans group might be older than the two other groups. Also is there a limitation for veterans depending on the time between TBI incident and enrolment?
In addition, are they going to be gender-matched?
Author response: The rationale for the selection of the three groups is now provided in Lines 167-171 in the marked copy and 163-167 in the clean copy . We have also explained in Lines 162-163 in the marked copy and 158-159 in the clean copy that control participants will be gender matched to the acute TBI group. We acknowledge in the limitations section that the time between TBI incident and veteran enrolment is something that we will be unable to control and meet our recruitment targets, as we can neither control the age of veterans nor the age at which patients suffer a TBI. To mitigate the likely older age in the veterans cohort, we will also seek to recruit recent service leavers through military TBI services.
Comment 2: Also it is better to clarify whether the control group is from the general population or veteran with no TBI as this affects the research question.

Author response: Most of our control group will be from the general population, as there is no specific pool of uninjured veterans from which to recruit a veterans control group. We accept that the control group is therefore predominantly a control for the acute TBI group, to which we will also compare the veterans group. However, we believe there is no reason to exclude veterans with no history of TBI.

Comment 3: Also, the authors should consider analyzing potential biomarkers depending on TBI stage and their progression with time.
Author response: We do intend to investigate this as part of the 12 month follow up and detect changes in biomarkers over the follow up period (indicated in Table 2).

Comment 4: In addition, how are you going to ensure adherence, address low enrolment and other methodological problems?
Author response: Adherence is less of an issue in an observational study than an interventional or drug trial, although we remain dependent on patient willingness to participate and will undertake an intention-to-treat style analysis based on enrolled subjects – now clarified in lines 235-236 in the marked copy and 230-231 in the clean copy. We have extensive experience of recruiting patients with TBI and have already started (successfully) recruiting.
We have modified the protocol to allow a greater pool of potential veterans, as we were struggling to recruit to this group as most interested patients had suffered mild rather than moderate or severe TBI and we have therefore broadened the veterans cohort to include mild TBI. Other methodological problems will be addressed as and when they arise, but we have not pre-specified specific responses.

Comment 5: In addition, it would be interesting to look for neurotransmitters changes and discuss their potential clinical value. https://www.nature.com/articles/s41380-018-0239-6
Author response: We will have the capacity to look for some neurotransmitter changes in serum of patients in our study, but the difficulty is in choosing the appropriate one or ones to analyse within budgetary constraints. We agree that there may be benefit and will look at this once our primary biomarkers have been tested.

Comment 6: Overall, the study covers an interesting topic though the study protocol lacks important details.
Author response: We hope that the revised version of our manuscript covers the important details that were lacking in our initial submission.

VERSION 2 – REVIEW

**REVIEWER NAME**	Lindblad, Caroline
**REVIEWER AFFILIATION**	Karolinska Institute, Clinical Neuroscience
**REVIEWER CONFLICT OF INTEREST**	No competing interests.
**DATE REVIEW RETURNED**	11-Jul-2024

**GENERAL COMMENTS**	The authors here provide a revision to their previously submitted manuscript. My comments have been satisfactorily answered. My recommendation for this article is still "accept".

VERSION 2 – AUTHOR RESPONSE
